# PCSK9 Affects Astrocyte Cholesterol Metabolism and Reduces Neuron Cholesterol Supplying In Vitro: Potential Implications in Alzheimer’s Disease

**DOI:** 10.3390/ijms232012192

**Published:** 2022-10-13

**Authors:** Bianca Papotti, Maria Pia Adorni, Cinzia Marchi, Francesca Zimetti, Nicoletta Ronda, Giovanni Panighel, Maria Giovanna Lupo, Antonietta Vilella, Daniela Giuliani, Nicola Ferri, Franco Bernini

**Affiliations:** 1 Department of Food and Drug, University of Parma, 43124 Parma, Italy; 2 Department of Medicine and Surgery, University of Parma, 43125 Parma, Italy; 3 Department of Medicine, University of Padova, 35131 Padova, Italy; 4 Department of Biomedical, Metabolic and Neural Sciences, Center for Neuroscience and Neurotechnology, University of Modena and Reggio Emilia, 41125 Modena, Italy

**Keywords:** PCSK9, cholesterol, brain, astrocyte, neuron, apolipoprotein E, Alzheimer’s disease, beta amyloid, neurotoxicity

## Abstract

The Proprotein Convertase Subtilisin/Kexin Type 9 (PCSK9) involvement in Alzheimer’s disease (AD) is poorly investigated. We evaluated the in vitro PCSK9 modulation of astrocyte cholesterol metabolism and neuronal cholesterol supplying, which is fundamental for neuronal functions. Moreover, we investigated PCSK9 neurotoxic effects. In human astrocytoma cells, PCSK9 reduced cholesterol content (−20%; *p* < 0.05), with a greater effect in presence of beta amyloid peptide (Aβ) (−37%; *p* < 0.01). PCSK9 increased cholesterol synthesis and reduced the uptake of apoE-HDL-derived cholesterol (−36%; *p* < 0.0001), as well as the LDL receptor (LDLR) and the apoE receptor 2 (ApoER2) expression (−66% and −31%, respectively; *p* < 0.01). PCSK9 did not modulate ABCA1- and ABCG1-cholesterol efflux, ABCA1 levels, or membrane cholesterol. Conversely, ABCA1 expression and activity, as well as membrane cholesterol, were reduced by Aβ (*p* < 0.05). In human neuronal cells, PCSK9 reduced apoE-HDL-derived cholesterol uptake (−41%; *p* < 0.001) and LDLR/apoER2 expression (*p* < 0.05). Reduced cholesterol internalization occurred also in PCSK9-overexpressing neurons exposed to an astrocyte-conditioned medium (−39%; *p* < 0.001). PCSK9 reduced neuronal cholesterol content overall (−29%; *p* < 0.05) and increased the Aβ-induced neurotoxicity (*p* < 0.0001). Our data revealed an interfering effect of PCSK9, in cooperation with Aβ, on brain cholesterol metabolism leading to neuronal cholesterol reduction, a potentially deleterious effect. PCSK9 also exerted a neurotoxic effect, and thus represents a potential pharmacological target in AD.

## 1. Introduction

Proprotein Convertase Subtilisin/Kexin Type 9 (PCSK9) protein is widely recognized as the master regulator of plasma cholesterol levels, due to its capacity to degrade the hepatic LDL receptors (LDLr), thus impairing the clearance of LDL-C from plasma [1].

PCSK9 was firstly identified in the brain as neural apoptosis-regulated convertase−1 (NARC-1), and its role in neurogenesis and brain development has been highlighted [2,3]. More recently, an involvement of PCSK9 in neurodegenerative disorders has been suggested [4]. In this regard, we and others previously observed that the cerebrospinal fluid (CSF) of patients affected by Alzheimer’s disease (AD) displays higher concentrations of PCSK9 as compared to non-AD subjects [5,6]. Similarly, elevated PCSK9 expression was detected in AD patients’ frontal cortex, the brain region most involved in the disease [7]. However, the relationship between PCSK9 and AD is still largely unknown, as genetic studies have given opposite indications of the association between PCSK9 variants and AD predisposition [8,9,10].

A growing body of evidence supports the concept of an altered brain cholesterol metabolism and trafficking in AD [11,12,13] and its critical role in AD pathogenesis [14]. Strongly supporting this link is the apolipoprotein E, the most important carrier of cholesterol in the brain, that in its E4 isoform is a well-known risk factor for late-onset AD [15].

In the brain, PCSK9 may have a major impact on cholesterol metabolism, by reducing the expression of LDLR as well as of other members of the same family, such as the very-low-density lipoprotein receptor (VLDLR), the apolipoprotein E receptor 2 (ApoER2), and the LDL receptor-related protein 1 (LRP1) [16,17,18], which regulate brain cholesterol homeostasis [11,19]. Specifically, these receptors promote the apoE-mediated internalization of cholesterol by neurons, an essential process providing lipids to these cells in order to maintain their physiological functions, such as synaptogenesis, neurite outgrowth, and repair of damaged membranes [20]. Since adult neurons progressively lose their endogenous cholesterol synthesis capacity and do not contribute anymore to the overall cholesterol synthesis in the brain [21], the internalization of exogenous cholesterol from other cells, mainly astrocytes [22], remains their main source of cholesterol supply [21]. The transport of cholesterol from astrocytes to neurons occurs through peculiar lipoprotein particles, similar to plasma HDL [23]. Specifically, cholesterol and apoE newly produced by astrocytes are secreted and assembled in HDL-like particles which, after remodeling and maturation, finally deliver cholesterol to neurons through the apoE-recognizing receptors mentioned above [11,24]. 

We hypothesize that PCSK9, which we found to be increased in the CSF of AD patients [6], by degrading the neuronal apoE-recognizing receptors and possibly affecting other steps of the brain cholesterol transport, may reduce cholesterol supply to neurons, with potentially deleterious consequences for neuronal function and survival. 

Based on these premises, the aim of the present study was to evaluate in vitro the effect of PCSK9 on cholesterol metabolism in astrocytes and neurons, with particular attention to the overall impact on neuron cholesterol supply. In addition, we investigated the potential neurotoxic effect of PCSK9. To these aims, we utilized specific cell models. Since PCSK9 in vivo is physiologically expressed and secreted by neurons [2,17,25,26], we prepared PCSK9-overexpressing human neuronal cells. On the contrary, since PCSK9 expression in astrocytes has not been reported, we adopted an experimental condition reproducing the paracrine action of this secretory protein, as previously described in macrophages [27,28]. Moreover, since recent data reported an influence of amyloid-beta (Aβ), the peptide typically accumulating in the brains of AD patients [29], on neuron and astrocyte cholesterol metabolism (i.e., endogenous synthesis, cell cholesterol content, and cholesterol efflux [30,31]), we also evaluated the effect of PCSK9 in the presence of Aβ fibrils, in order to reproduce an AD-like condition in vitro.

## 2. Results

### 2.1. Influence of PCSK9 on Astrocyte Cholesterol Metabolism 

Since the cholesterol necessary for neurons is mainly provided by astrocytes, we first evaluated the impact of exogenous PCSK9 on astrocyte intracellular cholesterol content. In addition, since overall cellular cholesterol content is the result of several processes, such as endogenous synthesis, uptake, and efflux, we evaluated the mechanisms underlying the PCSK9 effect on astrocytes’ cholesterol content by measuring these parameters as well.

#### 2.1.1. Intracellular Cholesterol Content

**Figure 1:**
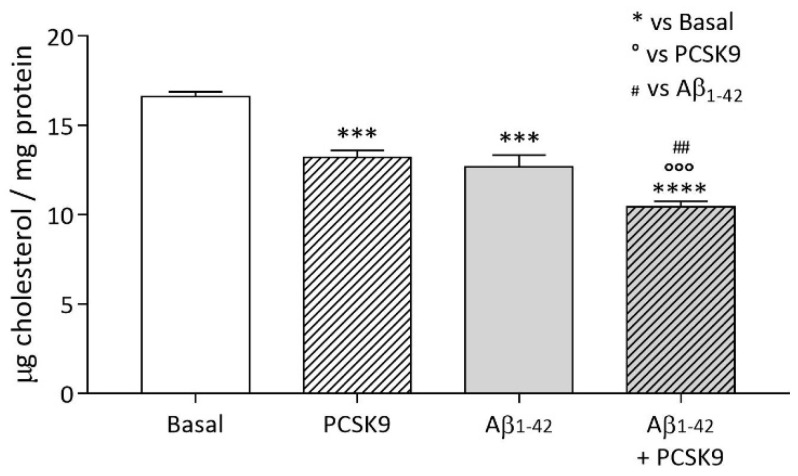
Influence of PCSK9 on intracellular cholesterol content in human astrocytes. U373 astrocytoma cells were treated with human recombinant PCSK9 [5 µg/mL] (striped bars) for 48 h, the last 24 of which with the addition of Aβ_1-42_ fibrils [1 µM] (grey bars), then intracellular cholesterol content was quantified through Amplex^®^ Red cholesterol fluorometric assay and protein content through BCA assay. Data are expressed as mean ± SD. Statistical analyses were performed using the one-way ANOVA test, with the post-hoc Tuckey’s multiple comparison test. A value of *p* < 0.05 was considered statistically significant. * refers to basal, ° to PCSK9-treated cells and ^#^ to Aβ_1-42_ fibrils-treated cells. ^##^ *p* < 0.01; *** and °°° *p* < 0.001, **** *p* < 0.0001.

As shown in Figure 1, treatment of U373 astrocytoma cells with PCSK9 determined a significant reduction in intracellular cholesterol content as compared to the basal condition (−20%; *p* < 0.001). Furthermore, we also found that the incubation with Aβ_1-42_ fibrils led to a reduction in cellular cholesterol content as compared to the basal condition (−24%; *p* < 0.001). The simultaneous incubation of both PCSK9 and Aβ_1-42_ led to a more marked drop in total cholesterol content (−37% compared to the basal condition, *p* < 0.0001; *p* < 0.001 compared to PCSK9 alone and *p* < 0.01 compared to Aβ_1-42_ alone). 

#### 2.1.2. Endogenous Cholesterol Biosynthesis

**Figure 2:**
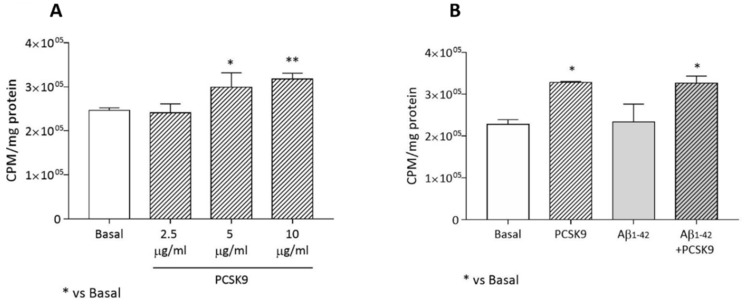
Influence of PCSK9 on endogenous cholesterol biosynthesis in human astrocytes. (**A**) U373 astrocytoma cells were treated for 48 h with increasing concentrations of human recombinant PCSK9, ranging from [2.5 µg/mL] to [10 µg/mL] (striped bars). (**B**) U373 astrocytoma cells were treated with human recombinant PCSK9 [5 µg/mL] (striped bars) for 48 h, the last 24 of which with the addition of Aβ_1-42_ fibrils [1 µM] (grey bars). Data are expressed as mean ± SD. Statistical analyses were performed using the one-way ANOVA test, with the post-hoc Tuckey’s multiple comparison test. A value of *p* < 0.05 was considered statistically significant. * *p* < 0.05; ** *p* < 0.01.

As reported in Figure 2A, PCSK9 incubation led to a dose-dependent increase in endogenous cholesterol biosynthesis in astrocytes (r^2^ for dose-response = 0.82), with a significant increase starting from [5 µg/mL] (*p* < 0.05 vs basal). This parameter was not affected by the concomitant incubation with Aβ_1-42_ fibrils (Figure 2B). PCSK9 at 5 µg/mL was then used for all the experiments.

#### 2.1.3. Cholesterol Uptake from apoE-rHDL and Lipoprotein Receptors Expression

**Figure 3:**
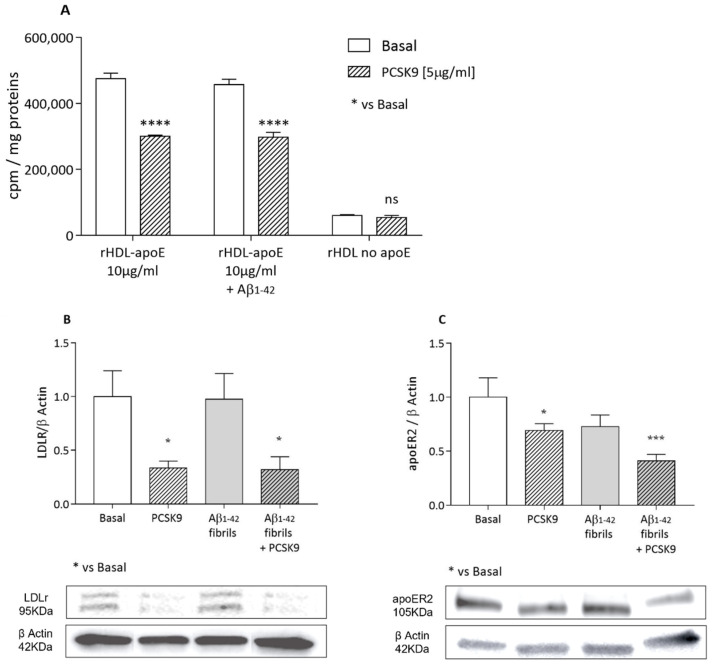
Influence of PCSK9 on cholesterol internalization in cultured human astrocytes. U373 cells were treated with human recombinant PCSK9 [5 µg/mL] (striped bars) for 48 h, the last 24 of which were with the addition of Aβ_1-42_ fibrils [1 µM] (grey bars). In order to evaluate apoE-rHDL internalization (**A**), cells were incubated for 4 h with radiolabeled rHDL-apoE; radioactivity was then quantified in cell monolayers. LDLR and apoER2 expression analysis (**B**,**C**) was performed through WB analyses. Data are expressed as mean ± SD. Statistical analyses were performed using the one-way ANOVA test, with the post-hoc Tuckey’s multiple comparison test. A value of *p* < 0.05 was considered statistically significant. * *p* < 0.05; *** *p* < 0.001; **** *p* < 0.0001.

Beyond endogenous synthesis, astrocytes are also able to physiologically obtain cholesterol by apoE-mediated internalization, thanks to the expression of the apoE-receptors [32,33]. We thus evaluated the impact of exogenous PCSK9 on this parameter. As reported in Figure 3A, a specific apoE-mediated cholesterol internalization from [^1-2,3^H] cholesterol-rHDL was observed. Recombinant PCSK9 led to a significant and marked reduction in cholesterol uptake mediated by these particles as compared to basal conditions (−36%, *p* < 0.0001). This parameter was not influenced by the pre-treatment with Aβ_1-42_ fibrils. Consistent with the specificity of apoE-mediated cholesterol internalization, a very low rate of cholesterol uptake was detected when rHDL particles without apoE were used as cholesterol donors, with no significant differences between the absence or presence of PCSK9 (Figure 3A). 

In accordance with the observed inhibitory effects of PCSK9 on specific apoE-mediated cholesterol internalization, LDLR expression (Figure 3B) was dramatically lowered following the incubation with exogenous recombinant PCSK9 (−66%; *p* < 0.05). A moderate, but still significant reduction in apoER2 expression (−31%, *p* < 0.05) was also observed following PCSK9 treatment (Figure 3C). On the other hand, the incubation with Aβ_1-42_ fibrils did not further affect the expression of these two receptors (Figure 3B,C). 

#### 2.1.4. Cholesterol Efflux

**Figure 4:**
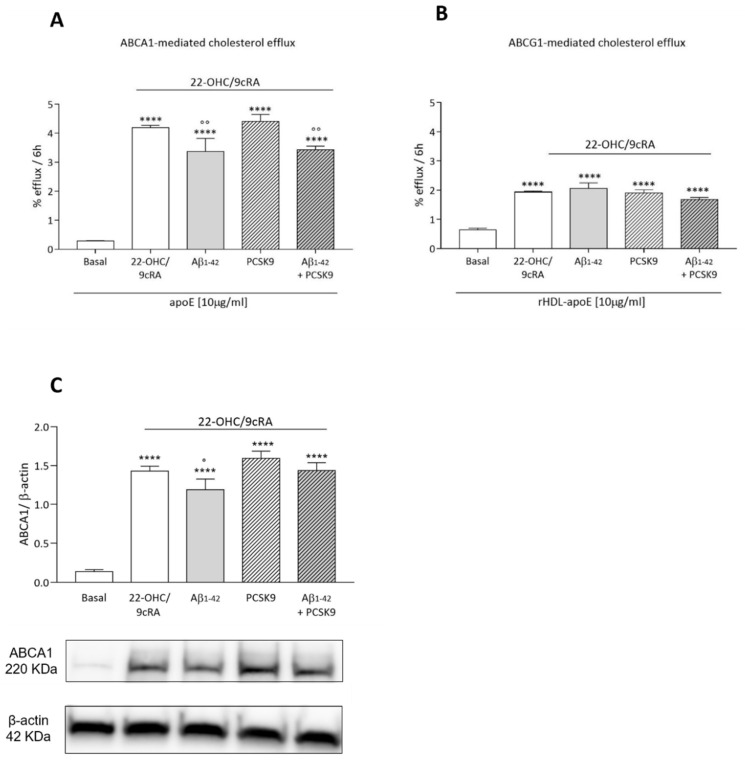
Influence of PCSK9 on cholesterol efflux in cultured human astrocytes. U373 cells were radiolabeled and treated with human recombinant PCSK9 [5 µg/mL] (striped bars) for 48 h, the last 24 h of which with the co-incubation of Aβ_1-42_ fibrils [1 µM] (grey bars) and 22-OHC/9cRA. In order to evaluate the cellular cholesterol efflux, exogenous apoE [10 µg/mL] and apoE-rHDL [10 µg/mL] were added for 6 h as cholesterol acceptors (**A**,**B**). Protein expression (**C**) was analyzed through WB analyses. Data are expressed as mean ± SD. Statistical analyses were performed using the one-way ANOVA test, with the post-hoc Tuckey’s multiple comparison test. A value of *p* < 0.05 was considered statistically significant. * refers to basal and ° to 22-OHC/9cRA-treated cells. ° *p* < 0.05; °° *p* < 0.01 **** *p* < 0.0001.

We then evaluated the impact of PCSK9 on cholesterol efflux from astrocytes, the first step of cholesterol transfer to neurons. In this process, astrocyte cholesterol is exported to the extracellular milieu by various membrane transporters, such as ABCA1 and ABCG1. Lipid-free apoE, as well as synthetic apoE-rHDL, resembling HDL-like particles identified in CSF, was used as an extracellular cholesterol acceptor, due to its ability to promote cholesterol efflux preferentially through ABCA1 and ABCG1, respectively [34,35]. PCSK9 did not affect ABCA1- and ABCG1-mediated cholesterol efflux, either in basal conditions or upon upregulation by LXR/RXR agonists 22OHC/9cRA (Figure 4A,B). On the contrary, Aβ_1-42_ fibrils significantly reduced ABCA1-mediated cholesterol efflux in 22-OHC/9cRA-stimulated U373 cells (−14%, *p* < 0.01; Figure 4A). Data on protein expression confirmed the lack of effect of PCSK9 on ABCA1 levels (Figure 4C), while Aβ_1-42_ fibrils treatment led to a modest, but significant, reduction in ABCA1 expression (*p* < 0.05). Aβ_1-42_ fibrils showed, instead, no impact on ABCG1 expression ([36]).

Since the cellular function is modulated not only by cholesterol content but also by its distribution [37,38], we also evaluated the impact of PCSK9 on membrane cholesterol content, a pool of cellular cholesterol strictly dependent on the ABCA1 activity [37]. In 22-OHC/9cRA-stimulated cells, PCSK9 did not significantly affect membrane cholesterol content (58 ± 5% membrane cholesterol/total cellular cholesterol in PCSK9-treated cells vs 53 ± 5% membrane cholesterol/total cellular cholesterol in non-PCSK9-treated cells; *p* = 0.37; [36]). Instead, the incubation with Aβ_1-42_ fibrils led to a significant decrease in plasma membrane cholesterol content (−11%; *p* < 0.05; [36]).

### 2.2. Influence of PCSK9 on Neuron Cholesterol Supply

Cholesterol supply to neurons is the final step of the HDL-mediated brain cholesterol transport, to guarantee the maintenance of the optimal intraneuronal lipid pool [23]. We have thus measured cholesterol internalization in control and PCSK9-overexpressing SH-SY5Y neuroblastoma cells, using as a cholesterol donor reconstituted apoE-rHDL, similar to that identified in the CNS, as well as astrocyte conditioned medium, containing particles directly secreted by PCSK9-treated astrocytes. 

#### 2.2.1. Cholesterol Uptake by apoE-rHDL

**Figure 5:**
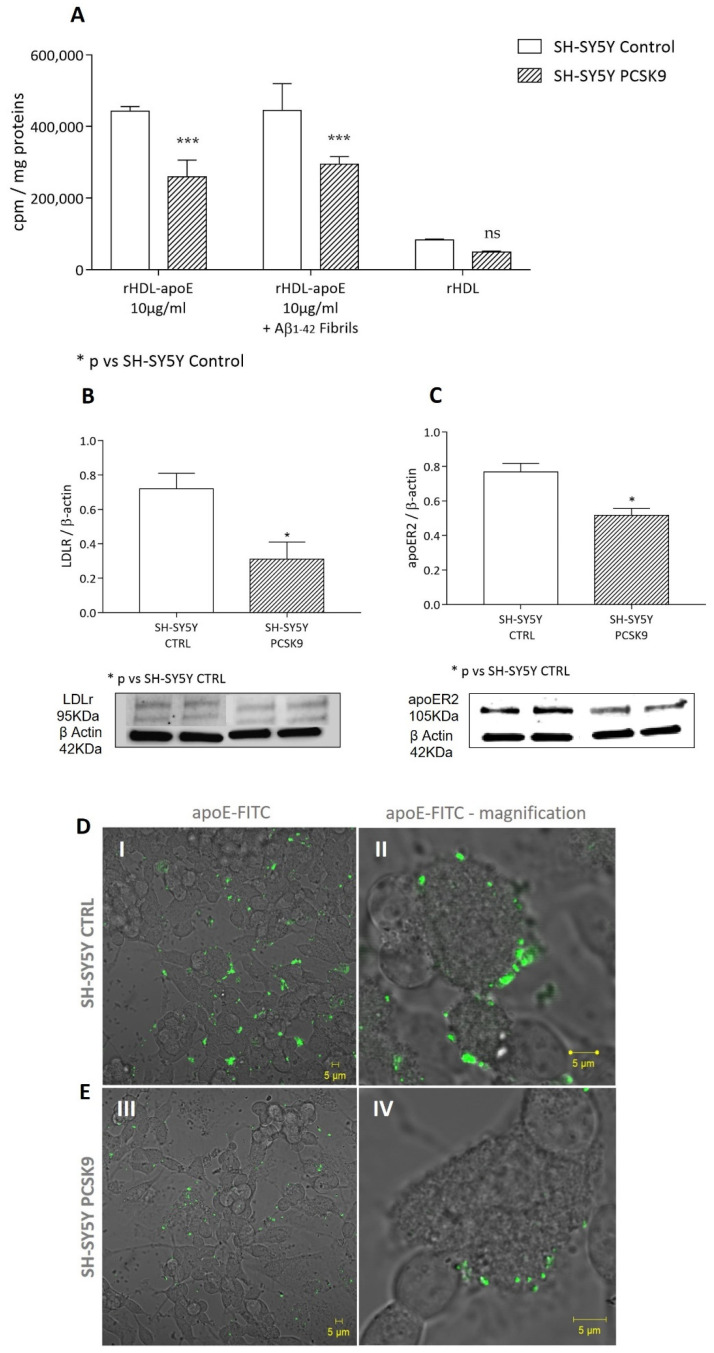
Influence of PCSK9 on cholesterol internalization from rHDL-apoE in human neurons. Differentiated SH-SY5Y control (plain bars) or PCSK9-overexpressing (striped bars) cells were treated with or without Aβ_1-42_ fibrils [1 µM] for 24 h. In order to evaluate cholesterol internalization (**A**), cells were then incubated for 4 h with radiolabeled rHDL either containing or not containing apoE; radioactivity was then quantified in cell monolayers. LDLR and apoER2 expression analysis (**B**,**C**) was performed through WB analyses. Data are expressed as mean ± SD. The interaction of apoE-FITC (in green) with living SH-SY5Y neuroblastoma cells control (**D**(**I**,**II**)) and PCSK9-overexpressing (**E**(**III**,**IV**)) conditions was monitored through confocal laser scanning microscopy, 40 min after apoE-FITC incubation. Statistical analyses were performed using the two-way ANOVA test, with the post-hoc Sidak’s multiple comparison test. A value of *p* < 0.05 was considered statistically significant. * *p* < 0.05; *** *p* < 0.001.

As reported in Figure 5A, following the exposure to [^1-2,3^H] cholesterol-apoE-rHDL, PCSK9-overexpressing cells showed a significantly lower cholesterol uptake as compared to control cells (−41%; *p* < 0.001). The reduction in cholesterol uptake occurred irrespective of the incubation of cells with Aβ_1-42_ fibrils. A similar effect, albeit less marked, was observed in SH-SY5Y control cells exposed to human exogenous PCSK9 as compared to non-treated cells (−16%, *p* < 0.05; [36]). 

As observed in astrocytes, consistent with the specificity of apoE-mediated cholesterol internalization in neurons, rHDL not containing apoE mediated a very low rate of cholesterol internalization as compared to apoE-rHDL particles, with no significant differences between PCSK9-overexpressing and control cells. 

Similar to astrocytes, we found that both the LDLR and apoER2 expression levels were significantly lower in PCSK9 overexpressing cells compared to control cells (*p* < 0.05, Figure 5B,C, respectively). 

Consistently, as shown in Figure 5D(I,II), the binding of FITC-labelled apoE (apoE-FITC) to the cell membrane was clear in control cells, with fluorescence distributed along the cell membrane. This phenomenon was less evident in the SH-SY5Y PCSK9 expressing cells (Figure 5E(III,IV)).

#### 2.2.2. Cholesterol Uptake by Astrocyte-Conditioned Medium 

**Figure 6:**
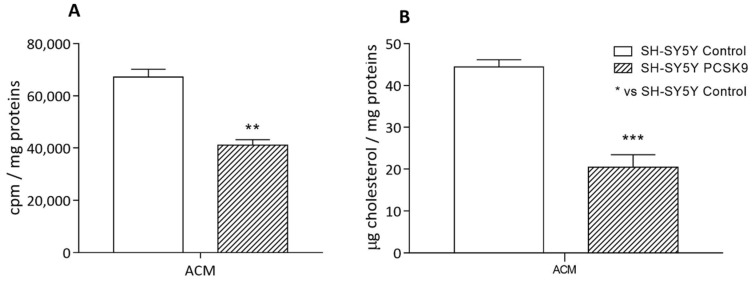
Influence of PCSK9 on cholesterol internalization from ACM in human neurons. Differentiated SH-SY5Y control (plain bars) or PCSK9-overexpressing (striped bars) cells were incubated for 24 h with ACM collected from radiolabeled (**A**) or not radiolabeled (**B**) U373 astrocytes (for details, see Methods section). Cell monolayers were then analyzed for their radiolabeled cholesterol content (**A**) and for their total cholesterol content (**B**). Data are expressed as mean ± SD. Statistical analyses were performed using the unpaired Student’s *t*-test. A value of *p* < 0.05 was considered statistically significant. ** *p* < 0.01; *** *p* < 0.001.

We, therefore, evaluated the influence of PCSK9 on the overall astrocyte-to-neuron transport process. To this aim, we incubated SH-SY5Y cells, both control and PCSK9-overexpressing, with the conditioned medium (ACM) derived from [^1-2,3^H] cholesterol-labeled astrocytes which were pre-treated with PCSK9 and Aβ_1-42_ fibrils, as performed in Figure 3. As shown in Figure 6, a marked reduction in cholesterol supplying was detected in PCSK9-overexpressing SH-SY5Y cells exposed to ACM, evaluated both as changes in radioactivity (−30%, compared to control cells; *p* < 0.01; Figure 6A) and in the total amount of intracellular cholesterol (−39% compared to control cells; *p* < 0.001, Figure 6B).

#### 2.2.3. Intracellular Cholesterol Content

**Figure 7:**
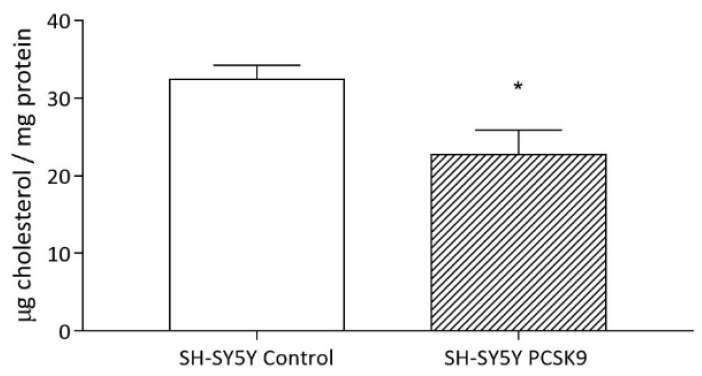
Influence of PCSK9 on intracellular cholesterol content in human neurons. Differentiated SH-SY5Y control (plain bars) or PCSK9-overexpressing (striped bars) cells were incubated for 24 h with culture medium supplemented with 1% *v*/*v* FCS. Cell monolayers were then analyzed for their total cholesterol content. Data are expressed as mean ± SD. Statistical analyses were performed using the unpaired Student’s *t*-test. A value of *p* < 0.05 was considered statistically significant. * *p* < 0.05.

We then evaluated whether the impaired uptake-induced PCSK9 would cause a modulation of the overall neuronal cholesterol content. As seen for astrocytes (Figure 1), PCSK9 overexpression also led to a significant reduction in intracellular cholesterol content in SH-SY5Y cells (−29%, *p* < 0.05; Figure 7). 

### 2.3. Influence of PCSK9 on Neurotoxicity

**Figure 8:**
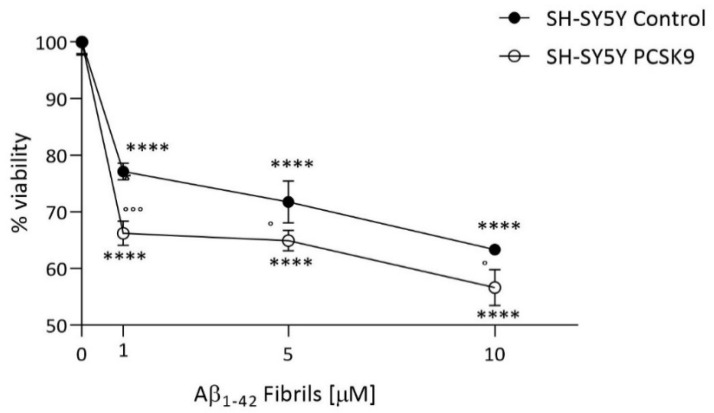
Influence of PCSK9 on cell viability in presence of Aβ_1-42_ fibrils. Differentiated SH-SY5Y neuroblastoma cells were treated for 48 h with a culture medium supplemented with 1% *v*/*v* of FCS (Basal) and with concentrations of Aβ_1-42_ fibrils, ranging from [1 µM] to [10 µM]. Cell viability was then assessed through the MTT assay. Data are expressed as mean ± SD. Statistical analyses were performed using the two-way ANOVA test, with the post-hoc Sidak’s multiple comparison test. A value of *p* < 0.05 was considered statistically significant. * refers to the Basal condition and ° refers to SH-SY5Y CTRL cells treated with the same concentration of fibrils. ° *p* < 0.05; °°° *p* < 0.001; **** *p* < 0.0001.

We finally tested the effect of PCSK9 overexpression on neuron susceptibility to cytotoxic stimuli. In particular, we evaluated the cell viability of SH-SY5Y in the presence of Aβ_1-42_ fibrils, a well-established in vitro model of neurotoxicity [39]. As shown in Figure 8, the incubation of control cells with Aβ_1-42_ fibrils dose-dependently lowered neuronal viability, with toxicity already detectable at [1 µM] (r^2^ for dose-dependency = 0.66). At all fibril concentrations, PCSK9-overexpressing cells displayed a markedly increased toxicity compared to control cells. Similar data were obtained using Aβ_1-42_ oligomers (see Figure S3). 

## 3. Discussion

Some evidence has suggested an involvement of PCSK9 in Alzheimer’s disease (AD), but the underlying mechanisms are still far from being understood. In this work, for the first time, we provide in vitro data suggesting that PCSK9 may affect the brain cholesterol metabolism, leading to impaired neuron cholesterol supply and reduced intracellular cholesterol content, with potentially important implications for AD development. Indeed, adult neurons strictly rely on exogenous cholesterol for their physiological functions, including those involved in the control of learning and memory [40,41]. In addition, we observed that PCSK9 potentiated neurotoxicity induced by the incubation with Aβ, the peptide typically depositing in AD brains. These data reinforce the previously well-established link between brain cholesterol metabolism dysfunction and AD pathogenesis [42], by pointing to PCSK9 as an additional contributor to the neuronal degeneration occurring in this disease. 

Astrocytes are the most abundant glial cells playing a relevant role in CNS by providing neurons with several trophic factors, including cholesterol [41,43]. We first observed that PCSK9 reduced the astrocytes’ cholesterol content, suggesting an unfavorable impact on the cholesterol pool from which transport to neurons originates. Exploring the possible mechanism involved in this reduction, we found that PCSK9 inhibited HDL-mediated cholesterol internalization through LDLR and apoER2. On the other hand, as reported for other cells [44], PCSK9 induced a dose-dependent increase in endogenous cholesterol synthesis, likely as a feedback response to cholesterol reduction mediated by the activation of the transcription factor Sterol Regulatory Element-Binding Protein (SREBP) [44,45]. Consistently, it has been reported that transcriptional control of cholesterol biosynthesis is relevant in astrocytes, as SREBP-1 is highly expressed [46]. Finally, contrary to what we have previously observed in macrophages [28], PCSK9 neither had an effect on the activity and expression of ABCA1 and ABCG1 nor on cholesterol distribution to the plasma membrane [37]. In astrocytes, overall, the reduced cholesterol uptake, the increased cholesterol synthesis, and the absence of a modulatory activity of PCSK9 on efflux result in a net reduction in the intracellular cholesterol content. Thus, our observations suggest that the increased synthesis is probably not able to compensate for the impaired uptake due to apoE receptor degradation. 

Interestingly, the ABCA1-mediated cholesterol efflux was reduced by Aβ_1-42_ fibrils, as a result of lower ABCA1 protein expression, and consequently reduced plasma membrane cholesterol. The inhibitory effect of Aβ_1-42_ on cholesterol efflux is in line with previous observations [23,47], highlighting a direct link between the increased amyloidogenesis in AD and brain cholesterol metabolism. However, our findings are in partial contrast with those of Azizidoost S. and colleagues. They observed an inducing effect of Aβ_1-42_ on ABCA1 protein levels, associated with a reduction in cholesterol efflux [30]. The reason for the discrepancy between these and our observation is not clear, but it is worth mentioning that our efflux data are supported by consistent changes in ABCA1 protein expression and in the membrane cholesterol content. In addition, the two experimental conditions adopted by us and the previous work are not directly comparable. In fact, in the paper by Azizidoost S. and colleagues, mouse astrocytes as well as Aβ oligomers have been used, while we treated human astrocytoma cells with Aβ fibrils. Overall, our data support a synergistic effect that can develop between Aβ fibrils and PCSK9 in reducing brain cholesterol fluxes from astrocytes. The reduction in intracellular cholesterol content induced by Aβ fibrils, in absence of a reduced cholesterol synthesis or uptake from extracellular donors and in presence of a reduced efflux, is somehow surprising. One possible explanation is that Aβ may compete with cholesterol internalization through apoE-independent pathways, such as the fluid phase macropinocytosis, a receptor-independent mechanism that has been described to promote Aβ uptake in astrocytes [48]. Further studies, out of the scope of this manuscript, are required to clarify this issue. 

Similar to astrocytes, in neurons, we demonstrated that PCSK9 overexpression is associated with a significant reduction in cholesterol uptake. This occurred irrespective of the presence of Aβ and led to an overall reduction in the intraneuronal cholesterol content. The PCSK9-dependent reduction in cholesterol uptake is, once again, clearly explained by the strong reduction in both LDLR and apoER2 expression, affecting the interaction between apoE and cells, as confirmed by in vitro live imaging through confocal microscopy. Notably, the degrading effect of PCSK9 on apoE-receptors has been previously reported [18,49,50], although not by all authors [51]. The reduction in neuron cholesterol uptake and content induced by PCSK9 was confirmed after the incubation of cells with a conditioned medium from PCSK9 and Aβ treated astrocytes. This result provides direct evidence of PCSK9 interference with cholesterol transport from astrocytes to neurons in vitro, with consequent neuronal cholesterol depletion.

Finally, in neurons, PCSK9 overexpression led to a worsening of neurotoxicity induced by different aggregation forms of Aβ, which have previously been demonstrated to have toxic effects in these cells [31,52]. These data clearly suggest a negative impact of PCSK9 on neuronal viability, further indicating a critical role in AD-related neurodegeneration. This activity may either relate to a direct effect on cell survival or a consequence of PCSK9-mediated cell cholesterol depletion. In line with the first hypothesis, a pro-apoptotic effect of PCSK9 was reported [26,53]. Accordingly, PCSK9 inhibition was shown to reduce neuronal apoptosis [54]. On the other hand, the cholesterol depletion hypothesis is in full accordance with the results of in vivo animal studies, in which silencing of lipoprotein receptors, implicated in cholesterol uptake and target of PCSK9, led to cognitive impairment [55,56,57,58,59], which was likely related to neuronal loss. Notably, as reported by Oliveira J. et al., the cognitive impairment observed in LDLr^−/−^ mice was associated with neuronal apoptosis in brain regions related to memory formation [58]. 

## 4. Materials and Methods

### 4.1. Cell Lines

In order to evaluate the impact of PCSK9 on cerebral cholesterol metabolism, the following cell models have been used: (I) human glioblastoma astrocytoma U373-MG cell line, as an in vitro standard surrogate model for human astrocytes; (II) human neuroblastoma SH-SY5Y cell line control, retrovirally transduced to overexpress human PCSK9, as an in vitro standard surrogate model of neurons. Both U373-MG astrocytoma and SH-SY5Y neuroblastoma control cells were kindly donated by Prof. Ovidio Bussolati from the Department of Medicine and Surgery of the University of Parma (Italy). In the SH-SY5Y cell line, PCSK9 overexpression has been obtained as described in the supplementary data and verified through gene and protein expression analyses, as well as by verifying the increased enzyme’s secretion in culture media (see Figure S1). Both cell lines were authenticated before beginning the experiments, through the Cell Line Authentication (CLA) service (Eurofins Genomic, Ebersberg, Germany). Control and PCSK9-overexpressing SH-SY5Y neuroblastoma cells were induced to differentiate into neuronal-like cells, following the incubation with all-trans-retinoic acid (ATRA; Sigma Aldrich, St. Louis, MO, USA) 10 µM for 6 days, in order to promote metabolic and morphological changes to mimic neuronal responses, prior to performing the experiments [60]. 

### 4.2. ApoE-Containing Reconstituted HDL

Reconstituted HDL containing apoE (apoE-rHDL), resembling the particles present in the human CSF, were prepared using the cholate dialysis procedure according to previous methods [61,62]. It contained ApoE: lecithin as a phospholipid (PL): cholesterol (molar ratio 1:100:2), with a final apoE concentration of 10 µM. Moreover, 70 µL of [^1-2,3^H] cholesterol (Perkin Elmer, Waltham, MA, USA) with a specific activity of 1mCi/mL were added to the preparation. Protein and cholesterol concentrations of the obtained particles were determined through the Pierce BCA Assay and Amplex^®^ Red cholesterol assay, respectively, according to the manufacturer’s instruction (Thermo Fisher Scientific, Carlsbad, CA, USA). In order to evaluate the apoE-independent uptake of cholesterol, liposomes made of lecithin and cholesterol (molar ratio 100:2) were prepared without adding apoE. Furthermore, in order to use apoE-rHDL as a cholesterol acceptor in the efflux experiments, an identical preparation without [^1-2,3^H] cholesterol was prepared as control. These particles were functionally characterized for their capacity to promote cholesterol efflux through specific pathways (see Figure S2) [63,64,65,66].

### 4.3. Astrocytes-Conditioned Medium (ACM)

In order to prepare ACM, U373 cells were seeded in DMEM with 10% FBS for 24 h and subsequently treated with or without 2 µCi/mL of [^1-2,3^H] Cholesterol in 1% FCS. Then, cells were treated with PCSK9 and Aβ_1-42_ as described in Section 4.6.2. ACM was then collected, cellular debris was removed by centrifugation, and the supernatant was transferred into a new sterile tube, which was kept at −20 °C until use [67].

### 4.4. Aβ_1-42_ Oligomers and Fibrils Preparation 

The Aβ_1-42_ oligomers and fibrils preparation procedure was carried out as previously described [68]. Briefly, the commercial Aβ_1-42_ peptide (Cayman Chemical, Ann Arbor, MI, USA) was resuspended in Hexafluoroisopropanol (HFIP; Sigma-Aldrich, St. Louis, MO, USA) to a final concentration of 1 mg/mL; HFIP was then allowed to evaporate under a nitrogen stream, and the resulting dried peptide film was resuspended in DMSO to a final concentration of 5 mM (Aβ stock solution). Subsequently, it was water-bath sonicated for 10 min. The Aβ_1-42_ oligomers were prepared by diluting the stock solution in a phosphate-buffered saline (PBS, Euroclone, Italy) solution to a final 100 µM concentration, before the incubation at 4 °C for 24 h. Aβ_1-42_ fibrils were prepared by diluting the Aβ stock solution in HCl 10 mM at pH 2 to a final 100 µM concentration, which was then incubated at 37 °C for 72 h. Atomic Force Microscopy (AFM) was used to verify the actual and correct formation of the Aβ_1-42_ fibrils ([36]) [68,69]. 

### 4.5. Cellular Viability Assay

Cell viability was evaluated through the MTT assay. Briefly, SH-SY5Y cells were treated with increasing concentrations of Aβ_1-42_ oligomers and fibrils (from 1 to 10 µM) in DMEM supplemented with 1% FCS (both from Euroclone, Italy) *v*/*v* for 48 h. The MTT assay was performed by incubating cells for 2 h at 37 °C with DMEM supplemented with 1% FCS and MTT [1 mg/mL]. After removing the supernatant, 300 µL of DMSO were added to each well to solubilize the newly-formed purple formazan. An aliquot of the suspension was finally collected and the absorbance was read at 570 nm. Cell viability was expressed as the percentage compared to the basal condition.

### 4.6. Cholesterol Metabolism Parameters

#### 4.6.1. Endogenous Cholesterol Biosynthesis

Endogenous cholesterol biosynthesis was evaluated by monitoring and quantifying the incorporation of a radioactive precursor ([^2–14^C] acetate) in the intracellularly synthesized cholesterol [44]. Briefly, U373 cells were incubated for 48 h with DMEM supplemented with 1% FCS and human recombinant PCSK9 [5 µg/mL], adding Aβ_1-42_ fibrils [1 µM] during the last 24 h; similarly, differentiated control and PCSK9-overexpressing SH-SY5Y cells were incubated with Aβ_1-42_ fibrils [1 µM] dissolved in DMEM + 1% FCS for 24 h. Then, cells were incubated for 24 h with DMEM supplemented with 0.5% *v*/*v* of sodium acetate, 0.2% *v*/*v* [^2–14^C] acetate and 0.4% *v*/*v* of FCS. Cell monolayers were washed with PBS and then lysed overnight with 0.1 M NaOH. Each sample was then saponified at 60 °C for 1 h, using alcoholic KOH and adding 105 counts per minute (cpm) of [^1,2-3^]H-cholesterol as an internal standard. Lipid extraction was then carried out using low-boiling petroleum ether; thin liquid chromatography (TLC) with a mobile phase composed of petroleum ether 40–60 °C/diethyl ether/acetic acid (70:30:1) was then performed in order to separate cholesterol from other sterols and to allow measurement of the radioactivity derived from the [^2–14^C] acetate through liquid scintillation counting. Data were expressed as cpm per milligram of protein, measured through the BCA assay, according to the manufacturer’s instructions. 

#### 4.6.2. Intracellular Cholesterol Content

Intracellular cholesterol content was evaluated by quantifying cholesterol in cell monolayers. The U373 cells were treated for 48 h with DMEM + 1% FCS and human recombinant PCSK9 [5 µg/mL], adding Aβ_1-42_ fibrils [1 µM] where required during the last 24 h. Control and PCSK9-overexpressing neuroblastoma cells were treated for 24 h with DMEM + 1% FCS. At the end of the treatments, cell monolayers were incubated with a 0.05 M Sodium chloride solution, 5 mM Cholic acid, 0.1% *v*/*v* TritonX-100, and 50 U/mL of Deoxyribonuclease I from bovine pancreas (Merck, Darmstadt, Germany) in order to promote cell lysis. The cholesterol content of each sample was finally quantified through the fluorescent Amplex^®^ Red Cholesterol assay kit following the manufacturer’s instructions. Data were expressed as µg of cholesterol over mg of the proteins in the cell monolayer, determined through the Pierce BCA Assay.

#### 4.6.3. Cholesterol Efflux

Cholesterol efflux from U373 astrocytoma cells has been evaluated through a radioisotopic technique. Specifically, U373 cells were radiolabeled with DMEM with 1% FCS, 2 µCi/mL of [^1-2,3^H] cholesterol, and an ACAT inhibitor compound (Sandoz; Merck, Darmstadt, Germany) at a concentration of 2 μg/mL to ensure all cholesterol was in free form. Subsequently, cells were treated for 48 h with human recombinant PCSK9 [5 µg/mL] and Aβ_1-42_ fibrils [1 µM], adding DMEM with 0.2% BSA, the ACAT-inhibitor, and the LXR/RXR agonists 22-hydroxycholesterol and 9-cis retinoic acid (22-OHC/9cRA) at [5 µg/mL] and [10 µM], respectively, in the last 18 h. Finally, cells were incubated for 6 h with DMEM supplemented with human recombinant lipid-free apoE [10 µg/mL] or apoE-rHDL [10 µg/mL], able to promote ABCA1- and ABCG1-mediated cholesterol efflux, respectively [34,62]. The medium was then collected and the radioactivity was quantified. A set of cell monolayers were washed with PBS before the incubation with cholesterol acceptors, in order to evaluate the amount of cholesterol inside the cells before the efflux phase. Cholesterol efflux was calculated as the percentage of radioactivity released into the medium over the total radioactivity incorporated by the cells [70].

#### 4.6.4. Cholesterol Uptake

Cholesterol internalization was evaluated by measuring the radioactivity in cell monolayers following the incubation with apoE-rHDL containing radiolabeled [^1-2,3^H] cholesterol [62,71]. Briefly, U373 were treated for 48 h with lipoprotein depleted serum (LPDS, Sigma-Aldrich, St. Louis, MO, USA) supplemented with human recombinant PCSK9 [5 µg/mL], adding Aβ_1-42_ fibrils [1 µM] in the last 24 h; similarly, LPDS-treated SH-SY5Y cells were incubated for 24 h with Aβ_1-42_ fibrils [1 µM]. Then, DMEM supplemented with apoE-rHDL, rHDL not containing apoE [10 µg/mL] or ACM was added for 4 (rHDL) or 24 (ACM) h at 37 °C. At the end of the incubations, cell monolayers were washed with PBS and then lysed for 18 h at 4 °C with NaOH 1N [62]. An aliquot of each sample was finally collected to quantify the radioactivity. Results were expressed as cpm over the total protein content, measured through the BCA assay. In the case of incubation of non-radiolabeled ACM, cell monolayers were lysed with 0.05 M sodium chloride, 5 mM Cholic acid, 0.1% *v*/*v* TritonX-100 and 50 U/mL of Deoxyribonuclease I, then intracellular cholesterol was quantified through the Amplex^®^ Red Cholesterol assay. Results were expressed as µg of cholesterol over the total protein content, measured through the BCA assay.

### 4.7. Western Blot Analyses

Protein expression in U373 and SH-SY5Y cells was analyzed through WB analyses. Specifically, ABCA1 and ABCG1 transporters’ expression was evaluated in cells treated as described in Section 4.6.3., while LDLR and apoER2 expression was analyzed in cells treated as described in the Section 4.6.4. Following the specific experimental treatments, cells were washed with ice-cold PBS. Cellular lysis was promoted by incubating cell monolayers in ice with a solution composed of RIPA Buffer (pH 7.4), supplemented with a cocktail of protease and phosphatase inhibitors (Sigma-Aldrich, St. Louis, MO, USA). The protein concentration in each sample was determined through the Pierce BCA Assay. Protein samples and a molecular mass marker were separated using 4–20% SDS-PAGE (Bio-Rad, Hercules, CA, USA) under denaturation and reduction conditions. Proteins embedded in the SDS-PAGE were then transferred into a polyvinylidene difluoride (PVDF) membrane (Bio-Rad, Hercules, CA, USA) and nonspecific binding sites were blocked in tris-buffered saline-Tween 20 (TBS-T 20) containing 5% non-fat dried milk (NFDM). Primary antibodies were diluted in TBS-T supplemented with 1% NFDM and incubated overnight at 4 °C under a mild shacking (anti-ABCA1, Novus Biological, Centennial, CO, USA; dilution 1:500 for anti-LDLR and anti-apoER2; anti-β Actin, Sigma-Aldrich, St. Louis, MO, USA; dilution 1:40,000). Membranes were washed three times with TBS-T and furtherly incubated for 1 h at room temperature with secondary antibodies (Bethyl Laboratories, Montgomery, TX, USA; dilution 1:10,000), preliminarily diluted in TBS-T supplemented with 1% NFDM. Immunoreactive bands were then detected by incubating the membranes with freshly reconstituted ECL reagent (Thermo Fisher Scientific, Waltham, MA, USA) and visualized using the ChemiDoc (BioRad, Hercules, CA, USA) chemiluminescent imaging system. Protein quantification was finally obtained by measuring the pixels in each band through ImageJ Fiji software [72].

### 4.8. Confocal Microscopy

Control and PCSK9-overexpressing SH-SY5Y cells were seeded on a coverslip fitting in a sterile flow chamber, then treated with a culture medium supplemented with 1.5 mg/mL of apoE (Preprotech, London, UK), previously conjugated with fluorescein isothiocyanate (FITC, Sigma-Aldrich, St. Louis, MO, USA) and purified by chromatography on a Sephadex G25 column in order to remove unbound FITC. The interaction between apoE-FITC and living cells was visualized by confocal microscope LSM 510 META Zeiss (objective 63X), equipped with a micro-incubator located on the microscope stage for 40 min after cell treatment. 

### 4.9. Statistical Analyses

Statistical analyses were performed using Prism 7 software (GraphPad Software, San Diego, CA, USA). Values are reported as mean ± standard deviation (SD) of experimental conditions performed in triplicate. Each result is representative of at least two independent experiments. Statistically significant differences among the mean values of each experimental group were investigated using the Student’s *t*-test, the one-way (for experiment in U373 cells) or two-way (for experiments on SHSY5Y control and PCSK9-overexpressing cells) analysis of variance (ANOVA), followed by the post-hoc Tuckey’s or Sidak’s multiple comparison tests, respectively. A value of *p* < 0.05 was considered statistically significant.

## 5. Conclusions

**Figure 9:**
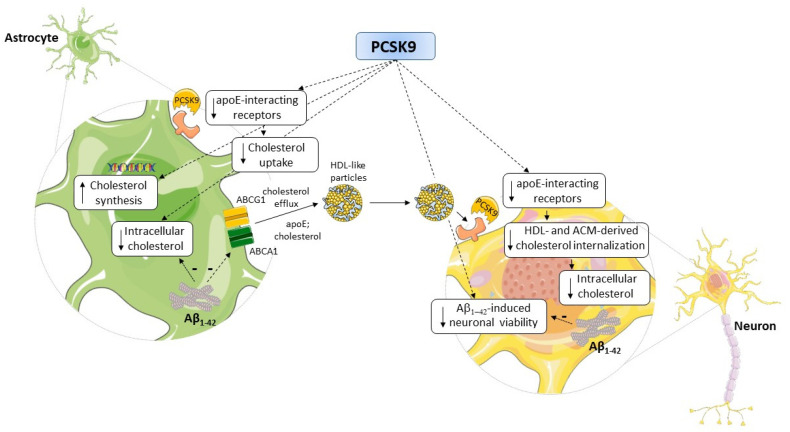
Impact on PCSK9 on cholesterol supplying to neurons and on neurotoxicity. Abbreviations: ABC: ATP-binding cassette; Aβ_1-42_: Amyloid beta fibrils fragment 1-42; ACM: astrocyte-conditioned medium; apoE: apolipoprotein E; HDL: high-density lipoprotein; PCSK9: Proprotein convertase subtilisin-Kexin type 9. Pictures were created by combining images from Smart Servier Medical Art (https://smart.servier.com, accessed on 9 September 2022). Servier Medical Art by Servier is licensed under a Creative Commons Attribution 3.0 Unported License (https://creativecommons.org/licenses/by/3.0/, accessed on accessed on 9 September 2022).

Many aspects of brain cholesterol metabolism have been demonstrated to be defective in AD [11], associated with learning and memory deficits, neuronal dysfunction, and degeneration [41]. PCSK9, whose levels are increased in the CSF of AD patients [6], may represent an additional deleterious factor affecting brain cholesterol metabolism in astrocytes and neurons, as well as exacerbating Aβ-mediated neurotoxicity, as summarized in Figure 9. In this view, PCSK9 may represent an attractive pharmacological target in AD. According to this hypothesis, in animal models of cognitive impairment induced by a high-fat diet, the administration of a PCSK9 inhibitor was associated with a reduction in Aβ_1-42_-formation, microglial activation, hippocampal apoptosis, and lower cognitive decline, although the results were not consistent across the studies [73,74,75,76]. Recently, these findings were confirmed in a specific AD mice model, in which PCSK9 inhibition by a monoclonal antibody led to improved hippocampus-dependent memory performances, occurring through the downregulation of LRP1-mediated Aβ clearance [50]. Notably, the authors attributed this improvement to a peripheral effect of PCSK9, given the inability of the anti-PCSK9 antibodies to cross the BBB. In this context, our data on the direct effect of PCSK9 on cerebral cells are valuable because they may pave the way for the development of new anti-PCSK9 strategies, based, for example, on small lipophilic molecules accessing the CNS and directly acting on astrocytes and neurons. This novel pharmacological approach, focused on a “lipid hypothesis” for AD pathogenesis, would open new perspectives for AD, for which no treatments are yet available. 

## Data Availability

The data presented in this study are available on request from the corresponding author.

## Supplementary Materials

The following supporting information can be downloaded at: https://www.mdpi.com/article/10.3390/ijms232012192/s1.
