# Supplementary material for: PCSK9 Affects Astrocyte Cholesterol Metabolism and Reduces Neuron Cholesterol Supplying In Vitro: Potential Implications in Alzheimer’s Disease
Source: Int J Mol Sci. 2022 Oct 13;23(20):12192. doi: 10.3390/ijms232012192 (PMC9602670; doi:10.3390/ijms232012192)
Supplement: Supplementary file 1 [file ijms-23-12192-s001.zip › ijms-1953199-SI.pdf]

PCSK9 Affects Astrocyte Cholesterol Metabolism and Reduces Neuron Cholesterol Supplying In Vitro: Potential Implications in Alzheimer's Disease

Bianca Papotti [1], Maria Pia Adorni [2], Cinzia Marchi [1], Francesca Zimetti [1,*], Nicoletta Ronda [1], Giovanni Panighel [3], Maria Giovanna Lupo [3], Antonietta Vilella [4], Daniela Giuliani [4], Nicola Ferri [3] and Franco Bernini [1]

[1] Department of Food and Drug, University of Parma, 43124 Parma, Italy
[2] Department of Medicine and Surgery, University of Parma, 43125 Parma, Italy
[3] Department of Medicine, University of Padova, 35131 Padova, Italy
[4] Department of Biomedical, Metabolic and Neural Sciences, Center for Neuroscience and Neurotechnology, University of Modena and Reggio Emilia, 41125 Modena, Italy
* Correspondence: francesca.zimetti@unipr.it; Tel.: +39-0521906172

1. Supplementary methods

1.1 *Characterization of SH-SY5Y overexpressing human PCSK9*

Empty pBMN-IRES-puromycin retroviral vector or the same vector carrying human PCSK9 coding sequence under CMV promoter were used to generate, respectively, SH-SY5Y control cells and PCSK9 overexpressing SH-SY5Y^{PCSK9} human neuroblastoma cell lines, exploiting the HEK293-Phoenix (φNX-A), as previously described [27]. Total RNA was extracted with iScript RT-qPCR sample Prep Reagent by Bio-Rad, CA, USA (cod. 1708899) according to manufacturer's instruction. mRNA was reverse transcribed with Xpert cDNA Synthesis Kit by Grisp, Portugal (cod. GK80.0100) with the following thermal conditions: 25°C for 10 min, 50°C for 15 min, 85°C for 5 min, 4°C hold. The obtained cDNA was used for SYBR green-based qPCR analysis (ExcelTaq™ kit by SmoBio, Taiwan, cod. TQ1201) with the following thermal conditions: 95°C for 2min (1 cycle), 95°C for 15 sec and 60°C for 60 sec (40 cycles). Human PCSK9 primer sequences have been designed on human mRNA template (NM_174936.3) using Primer BLAST by NCBI and are: FWD 5′-CCTGCGCGTGCTCAACT-3′ and REV 5′-GCTGGCTTTTCCGAATAAACTC-3′. 18S was used as loading control and primer sequences have been designed with Primer BLAST against 18S template (X03205.1): FWD 5′-CGGCTACCACATCCACGGAA-3′ and REV 5′-CCTGAATTGTTATTTTTCGTCACTACC-3′. Reverse Transcription was performed on C100™ Touch Thermal Cycler by Bio-Rad, qPCR was performed on CFX96™ Real-Time System by Bio-Rad. Secreted PCSK9 was measured on conditioned media using the Human Proprotein Convertase 9/PCSK9 Quantikine® ELISA kit by R&D Systems, MN, USA (cod. DPC900) (intra-assay precision: CV% 5.43; inter-assay precision: CV% 4.76). GraphPad Prism v.8.2.1 was used for data analysis by generating a 4PL curve-fit. Before testing, media were cleared from cell debris by centrifugation (15.000 rpm at 4°C for 10 min) and tested undiluted. Intracellular PCSK9 protein content was evaluated by blotting 20μg of denaturated total proteins (anti-PCSK9 ab by GeneTex, CA, USA, cod. GTX129859, 1:1000), using β-actin as normalizing protein (Sigma-Aldrich, St. Louis, MO, cod. A5316, 1:5000). Total proteins were retrieved by lysing cell monolayers with a home-made lysis buffer (1% NP-40, 150 mM NaCl, 50 mM Tris-HCl at pH 7.5, 1% phosphate inhibitors, 1% protease inhibitors). Samples were cleared from cells debris by centrifugation (15.000 rpm at 4°C for 10 min) and quantified with the BCA assay (Serva, Germany, cod. 39228.02). Samples for blotting were prepared using a home-made Laemmli Buffer (5X recipe: 10% SDS, 25% 2-betamercaptoethanol, 50% glycerol, 0.01% bromophenol blue, 0.3125% Tris-HCl, pH 6.8), boiled at

95-100°C for 5 min, and loaded on 4% - 20% Mini-PROTEAN TGX Precast Protein Gels (Bio-Rad, cod. 4561094). Proteins were then transferred onto a nitrocellulose membrane (Trans-Blot Turbo Mini 0.2 μm Nitrocellulose Transfer Packs, Bio-Rad, cod. 1704158) using TransBlot Turbo™ Transfer System by Bio-Rad. The membrane was blocked for aspecific signals with a blocking solution (5% w/v non-fat milk in TBS-Tween 20 1X) and then challenged with the primary antibodies listed above (overnight, 4°C), followed by an incubation with the appropriate HRP-conjugated secondary antibodies (anti-rabbit: Jackson ImmunoResearch, PA, USA, cod. 113-036-045, 1:5000; anti-mouse: Jackson ImmunoResearch, cod. 115-036-062, 1:5000). C400 Azure system by Aurogene, Italy, was used to reveal chemiluminescent signals (ECL kit from Advansta, CA, USA, cod. K-12045-D50). Data elaboration was performed with ImageLab software by Bio-Rad.

1.2 Functional characterization of apoE-rHDL

ApoE-containing rHDL (apoE-rHDL) prepared as described in the Method section (main manuscript) were functionally characterized based on their ability to promote cholesterol efflux through the aqueous diffusion as well as through ABCA1 and ABCG1 transporters. Cholesterol efflux was evaluated through a radioisotopic technique, as previously reported [13]. Specifically, J774 murine macrophages in basal conditions were used to analyse the aqueous diffusion process, while J774 cells treated with cpt-cAMP (Sigma-Aldrich), able to upregulate ABCA1 transporter [63]; hABCG1-expressing Chinese hamster ovary (CHO)-K1 cells [64], were used to specifically investigate the ABCA1- and ABCG1-mediated cholesterol efflux pathway.

2. **Supplementary results**

2.1. Molecular characterization of transfected PCSK9-overexpressing neuroblastoma cells

Following the retroviral transfection, PCSK9-overexpressing SH-SY5Y neuroblastoma cells were characterized for the gene and protein expression of PCSK9, as well as for PCSK9 secretion in the media. As reported in **Figure S1A**, **S1C** and **S1D**, a marked induction of gene and protein expression of PCSK9 was observed in overexpressing SH-SY5Y compared to control cells, in which PCSK9 was barely detectable ($p<0.001$ and $p<0.01$, respectively). At the same manner, the protein secretion was significantly higher in differentiated SH-SY5Y PCSK9 compared to control cells in which the production of PCSK9 was negligible (**Figure S1B**; $p<0.0001$).

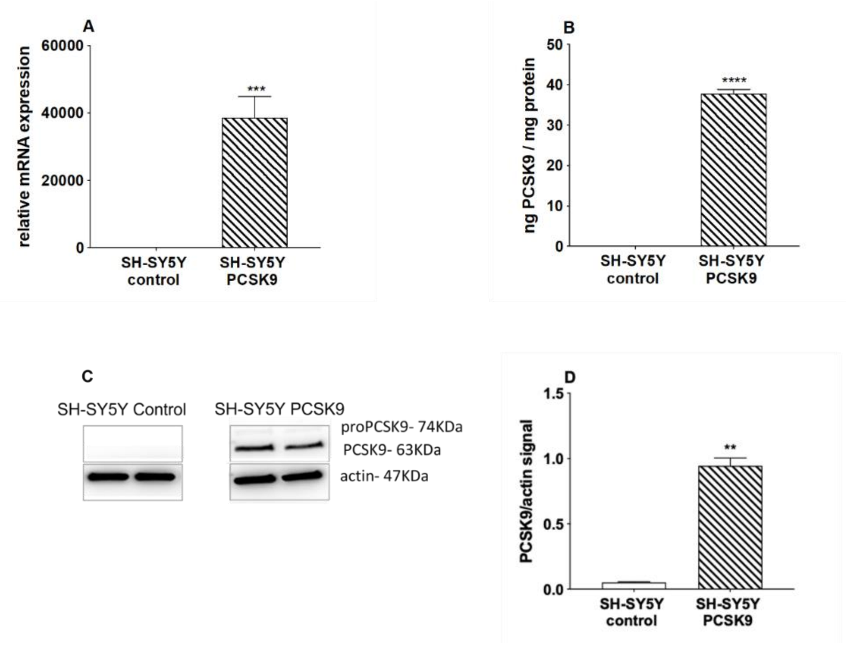

Figure S1. *Gene and protein expression and secretion of PCSK9 in SH-SY5Y neuroblastoma cells, control and overexpressing PCSK9*. **A:** PCSK9 mRNA levels have been analysed by Real-time PCR after cell transfection. **B:** PCSK9 secretion was performed in conditioned medium by ELISA kit. **C, D:** PCSK9 and proPCSK9 protein expression was evaluated by Western Blotting and furtherly quantified with ImageLab software. Data are expressed as means ± SD. Unpaired t-test was applied to compare the different means. A value of $p<0.05$ was considered statistically significant. **p<0,01, ***p>0.001 and ****p>0.0001 compared to relative control cells.

2.2. Functional characterization of apoE-rHDL

Newly synthesized apoE-rHDL were preliminarily tested for their capacity to promote cholesterol efflux through specific membrane transporters. Specifically, apoE-rHDL resembled HDL-like particles present in the CSF, were used as cholesterol acceptors, thus mimicking the cholesterol transport from astrocytes to neurons. According to the preparation method, we expected a size within the range of about 9nm [23], that has been previously demonstrated to promote cholesterol efflux selectively through the cholesterol transporter ABCG1 and the aqueous diffusion process, but not through the transporter ABCA1 [65]. Consistently, as reported in **Figure S2**, we found that the newly-synthesized apoE-rHDL [10 µg/ml] were able to produce a significant cholesterol efflux through the ABCG1 transporter as well as through the aqueous diffusion process, even in a more efficient manner compared to isolated plasma HDL. On the other hand, the apoE-rHDL were not able to promote cholesterol efflux through ABCA1 similarly to plasma HDL and differently from free apoA-I, the preferential acceptor for this cholesterol efflux pathway [65].

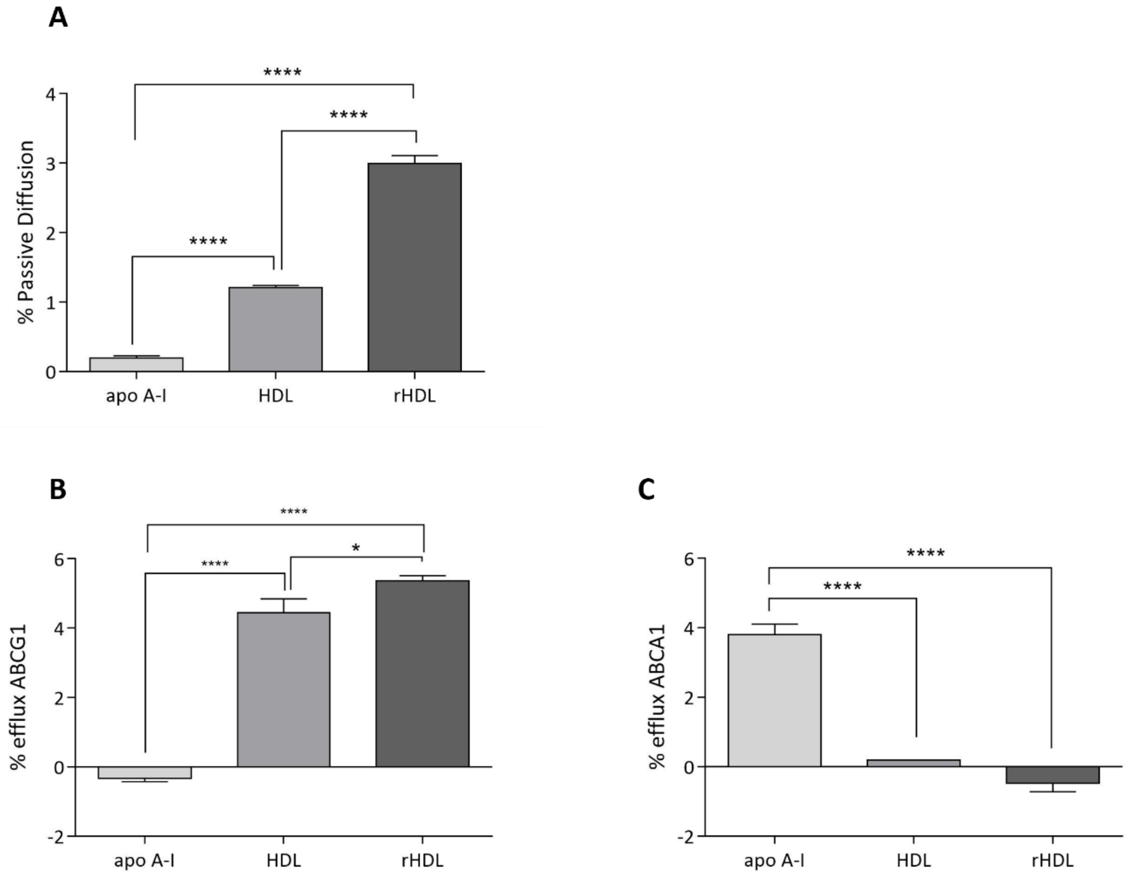

Figure S2. *Characterization of apoE-rHDL for the capacity to promote cell cholesterol efflux.* **Panel A**. cholesterol efflux capacity through aqueous diffusion; **Panel B** ABCG1-mediated cholesterol efflux capacity; **Panel C**. ABCA1 mediated cholesterol efflux capacity. Isolated human ApoA-1 and HDL were used as internal controls.

Data were performed in triplicate and are expressed as mean ± SD. Statistical analyses were performed using the ordinary one-way ANOVA test, with the post-hoc Tuckey's multiple comparison test. A value of $p<0.05$ was considered statistically significant. *$p<0.05$,**** $p<0.0001$.

2.3. Influence of PCSK9 on Aβ1-42 oligomers-induced neurotoxicity

Similarly to the evaluation of PCSK9 impact $A\beta_{1-42}$ fibrils-induced neurotoxicity (Main text, **Figure 8**), we tested the potential neurotoxic effect of PCSK9 in neuroblastoma cells exposed to $A\beta_{1-42}$ oligomers, an alternative Aβ aggregation form widely used as an in vitro model of neurotoxicity [5]. As shown in **Figure S3**, the also the incubation of control cells with $A\beta_{1-42}$ oligomers, similarly to the observations reported for $A\beta_{1-42}$ fibrils, dose-dependently lowered neuronal viability, reaching significance at [10μM] (r^2 for dose-dependency = 0.9). At all oligomer concentrations, PCSK9 overexpressing cells displayed a markedly increased toxicity compared to control cells.

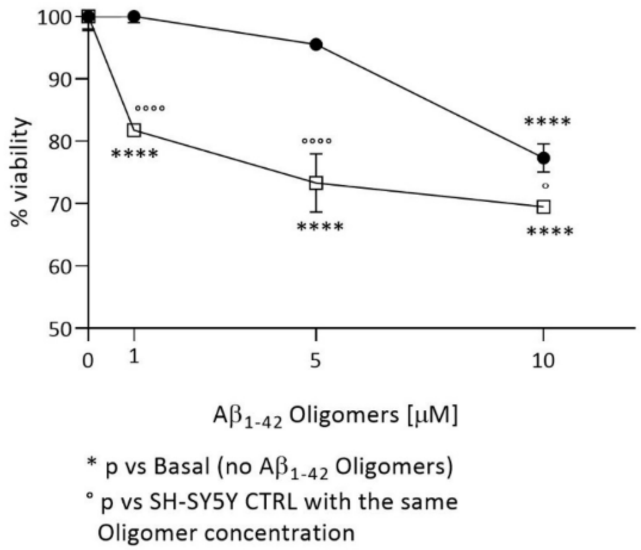

Figure S3. *Influence of PCSK9 on cell viability in presence of Aβ1-42 oligomers in human neurons.* Differentiated SH-SY5Y neuroblastoma cells were treated for 48 hours with a culture medium supplemented with 1% v/v of FCS (Basal) and with concentrations of $A\beta_{1-42}$ oligomers, ranging from [1μM] to [10μM]. Cell viability was then assessed through the MTT assay. Data are expressed as mean ± SD. Statistical analyses were performed using the Two-way ANOVA test, with the post-hoc Sidak's multiple comparison test. A value of $p<0.05$ was considered statistically significant. * refers to the Basal condition and ° refers to SH-SY5Y CTRL cells treated with the same concentration of oligomers. ° $p<0.05$; **** and °°°° $p<0.0001$.
